# Fliposomes: pH-Sensitive Liposomes Containing a *trans*-2-morpholinocyclohexanol-Based Lipid That Performs a Conformational Flip and Triggers an Instant Cargo Release in Acidic Medium

**DOI:** 10.3390/pharmaceutics3030379

**Published:** 2011-07-11

**Authors:** Nataliya M. Samoshina, Xin Liu, Barbora Brazdova, Andreas H. Franz, Vyacheslav V. Samoshin, Xin Guo

**Affiliations:** 1 Department of Pharmaceutics and Medicinal Chemistry, University of the Pacific, 751 Brookside Road, Stockton, CA 95211, USA; 2 Department of Chemistry, University of the Pacific, 3601 Pacific Ave., Stockton, CA 95211, USA

**Keywords:** pH-sensitive liposomes, fliposomes, polyethyleneglycol (PEG), conformational switch, controlled drug release, triggered release

## Abstract

Incorporation of a pH-sensitive conformational switch into a lipid structure enables a drastic conformational flip upon protonation that disrupts the liposome membrane and causes rapid release of cargo specifically in areas of increased acidity. pH-sensitive liposomes containing the amphiphile (**1**) with *trans*-2-morpholinocyclohexanol conformational switch, a phospholipid, and a PEG-lipid conjugate were constructed and characterized. The optimized composition—**1**/POPC/PEG-ceramide (50/4/5)—could be stored at 4 °C and pH 7.4 for up to 1.5 years, and was stable in blood serum *in vitro* after 48 h at 37 °C. Liposomes loaded with ANTS/DPX or methotrexate demonstrated an unusually quick content release (in a few seconds) at pH below 5.5, which was independent of inter-liposome contact. The pH-titration curve for the liposome leakage paralleled the curve for the acid-induced conformational flip of **1** studied by ^1^H-NMR. Freeze-fracture electron microscopy images showed budding and division of the bilayer at pH 5.5. A plausible mechanism of pH-sensitivity involves an acid-triggered conformational flip of **1**, shortening of lipid tails, and membrane perturbations, which cause the content leakage. The methotrexate-loaded liposomes demonstrated much higher cytotoxicity in HeLa cells than the free drug indicating that they can serve as viable drug delivery systems.

## 1. Introduction

pH-triggered release is an attractive approach to improving liposomes as drug/gene delivery systems because proton concentration is elevated under numerous physiological and pathological conditions including endosome processing, inflammation, ischemia, and solid tumor growth [1-13]. However, it is still a considerable challenge to develop liposomes that promptly respond to small variations of pH while preserving other attributes that are important for drug delivery such as sufficient stability in blood circulation and on shelf.

**Figure 1.**
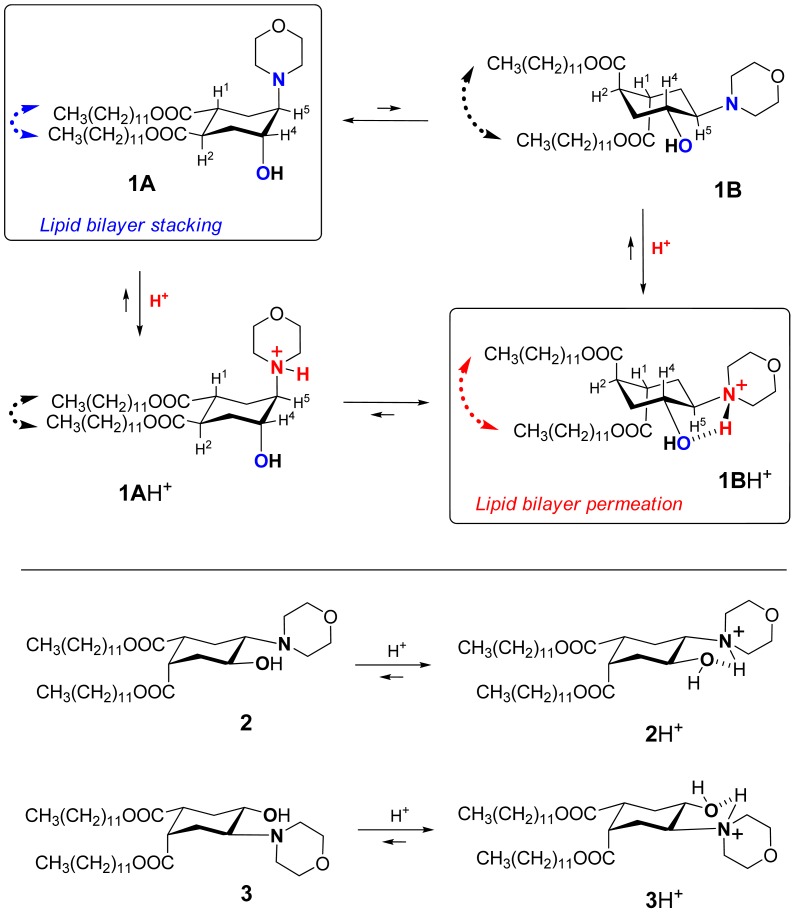
Protonation-induced conformational switch of lipid tails in amphiphile **1**, and an absence of such a switch in amphiphiles **2** and **3**.

Recently we suggested a novel strategy to render liposomes pH-sensitive: a protonation-induced conformational switch of lipid tails in the liposome membrane (for the preliminary communication see [14]). For proof of concept, an amphiphile **1** based on *trans*-2-morpholinocyclohexanol was introduced as a pH-sensitive conformational switch of liposome bilayer (Figure 1). The pH-induced flip of its chair conformation was demonstrated by NMR data [14]. In the original conformational equilibrium **1A ⇆ 1B** (Figure 1), the morpholine and the hydroxy groups of **1** were predominantly in axial positions while both lipidic *trans*-dodecyloxycarbonyl groups COOC_12_H_25_ adopted equatorial positions (**1A**). The protonation of the amine nitrogen upon addition of acid resulted in a strong hydrogen bond between the morpholine and the hydroxy groups, forcing both groups to adopt equatorial positions. This impulse was mechanically transmitted via the conformational flip of the cyclohexane ring to force both ester groups into axial positions (**1B**H^+^ on Figure 1), thereby drastically increasing the spatial separation of the lipid tails and perturbing the structure of bilayer. After incorporation into liposome membranes, lipid **1** enhanced significantly the release from both the traditional liposomes based on phospholipids and the sterically hindered liposomes containing mPEG2000-ceramide (PEG-ceramide) upon exposure to lowered pH [14].

This approach is different from the previous design of pH-sensitive liposomes in which peptides were used as conformational switches to induce leakage in acidic medium [13,15-17]. pH-sensitive synthetic peptides were added to a lipid composition in a very small quantity, at ratios varying from 1/30 to 1/300000 [13]. In our studies the pH-responsive amphiphile **1** constituted from 25% to 90% of the lipid composition. Apparently, the mechanism of triggering the leakage must be different. It is also different from the pH-sensitivity mechanism of the polymer-modified liposomes [2,7,18].

The preliminary results encouraged us to study in more details the physicochemical and biological properties of this novel type of liposomes including compatibility of the pH-sensitive conformational trigger with commonly used lipids, ability to control the rate and extend of liposome content release, mechanism of lipid membrane destabilization, and viability of liposomes for drug delivery. Of special interest is the ability of **1** to trigger the PEGylated liposomes due to their prolonged half life in blood circulation and successful applications in drug/gene delivery [7,19-23].

To address these issues, we designed, prepared and characterized a series of PEGylated liposomes equipped with the molecular device **1**. The pH-driven conformational flip of **1** was studied by ^1^H NMR titration. The pH-triggered release of the liposomes was measured using the ANTS/DPX fluorescent assay [16,24]. The mechanism of the acid-triggered lipid membrane destabilization was probed by freeze-fracture electron microscopy (FFEM) [25-28]. Selected lipid compositions were utilized to construct liposomes encapsulating a widely used anticancer drug methotrexate [29,30], followed by the characterization of their pH-triggered drug release by equilibrium microdialysis [31] and anticancer activity in HeLa cells (human cervical cancer).

## 2. Experimental Section

### 2.1. Materials

The lipids 1-palmitoyl-2-oleoyl-*sn*-glycero-3-phosphocholine (POPC), 1,2-dipalmitoyl-*sn*-glycero-3-phosphocholine (DPPC), 1-palmitoyl-2-oleoyl-*sn*-glycero-3-phosphoethanolamine (POPE), N-palmitoyl-sphingosine-1-(succinyl[methoxy(polyethylene glycol)2000]) (PEG-ceramide), 1,2-dipalmitoyl-*sn*-glycero-3-phosphoethanolamine-N-[methoxy(polyethylene glycol)-2000] (PEG-DPPE) were purchased from Avanti Polar Lipids, Inc. (Alabaster, AL, USA) and used without further purification. The fluorophores aminonaphthalene-1,3,6-trisulfonic acid disodium salt (ANTS) and *p*-xylene-bis-pyridinium bromide (DPX) were purchased from Invitrogen-Molecular Probes (Eugene, OR, USA). Methotrexate (MTX), detergent C_12_E_8_ (octaethyleneglycol monododecyl ether), the folate-deficient medium (FDMEM) and all other chemicals were purchased from Sigma-Aldrich or Fisher Scientific. Fetal bovine serum was obtained from Tissue Culture Biologicals (Tulare, CA, USA). HeLa cells were received from American Type Culture collection (Rockvile, MA, USA). CellTiter 96AQueous One Solution reagent was purchased from Promega (Madison, WI, USA). The 24-well and 96-well plates were obtained from TPP (Switzerland). The morpholinocyclohexanol-based amphiphiles **1**, **2** and **3** (Figure 1) were prepared according to the reported procedures [14] with modifications (see Supplementary Data).

### 2.2. NMR analysis of the pH-dependent conformational equilibrium of lipid 1

To characterize the pH-induced change of conformational equilibrium, the lipid **1** was dissolved in CD_3_OD (0.03 M), and the changes of its ^1^H NMR spectra (600 MHz) were monitored during titration of the solution with *d*-trifluoroacetic acid (*d*-TFA) and with a base—1,8-diazabicyclo[5.4.0]undec-7-ene (DBU). Acid or base were dissolved in CD_3_OD (1 M) and added to the solution of **1** in NMR tube (0.6 mL) in small portions (1–5 μL). After every addition the solution was thoroughly mixed by gentle shaking, and its pH (pD) was measured using a micro combination pH electrode for NMR cells (9826BN, Thermo Scientific). Shaking and measuring was repeated 3–4 times until a constant value of pD was achieved. After recording the ^1^H NMR spectrum, pD was measured again: the original and final values matched within 0.05 units. The spin-spin coupling constants between several couples of vicinal protons attached to the cyclohexane moiety are strongly conformation-dependent, which allowed an assignment of a predominant conformation and an estimation of a position of the conformational equilibrium (see section 3.1) as described previously [14,32-36].

### 2.3. Liposome preparation

Liposomes were prepared by the freeze-thawing method based on the procedure of Monnard and co-workers [37]. Both the purchased lipids (POPC, DPPC, POPE, PEG-ceramide and PEG-DPPE) and the synthesized lipids **1**–**3** were dissolved in dichloromethane before being used for liposome preparation. Ratios of lipid components in liposomes were in mole units. For each liposome preparation, appropriate amounts of lipids in dichloromethane solution were mixed in a Pyrex brand glass test tube (25 mm × 125 mm) to give 10 umol total lipids. Dichloromethane was evaporated under reduced pressure at room temperature (RT) to form a lipid film at the bottom of the tube. The lipid film was subjected to high vacuum for 5–6 h to remove residual solvent. The lipid film was then hydrated in 2 mL of buffer containing either the fluorophores ANTS/DPX (50 mM ANTS, 50 mM DPX, and 5 mM HEPES, pH 7.4) or the anticancer drug MTX (12.5 mM in 5 mM HEPES, pH 7.4) on a bench top vortex machine under argon. The lipid suspension was then frozen by submergence into acetone/dry ice, followed by melting in water bath. The freeze-thawing cycle was repeated ten times and the resultant liposome suspension with encapsulated contents (ANTS/DPX or MTX) was extruded eleven times through a 0.2-μm polycarbonate membrane (Nucleopore, Pleasanton, CA, USA) with a hand-held extrusion device (Avanti Polar Lipids, Alabaster, AL, USA). The extruded liposomes (the opaque fraction) were separated from the unencapsulated materials on a Sephadex G-75 size exclusion column equilibrated in a pH 7.4 buffer (5 mM HEPES and 145 mM NaCl). For storage, the liposome preparations were kept in darkness at 4 °C, under argon in sealed Pyrex glass tubes. Placebo liposomes that contained no encapsulated agents were also prepared as described above.

### 2.4. Physicochemical characterization of liposomes

The hydrodynamic diameter and the ζ-potential of the liposomes were measured with a Malvern Zeta 3000 dynamic light scattering instrument (Malvern Instruments Ltd., Worcester-shire WR14 1XZ, UK), using the automatic algorithm mode. POPC concentrations were determined by a lipid phosphorous assay based on the procedures of Bartlett [38].

The lipid composition of **1**/POPC/PEG-ceramide liposomes was confirmed by ^1^H NMR as we reported recently [39]. Briefly, aliquots (10 μmol total lipids in 2 mL) of the liposome preparations were evaporated to dryness in high vacuum. The lipid components of the residue were extracted by 4 × 0.25 mL CHCl_3_, evaporated and re-dissolved in 0.7 mL of CDCl3 or CDCl3-DMSO. The ^1^H NMR spectra of the solutions were acquired and the signature peaks of the lipid components were integrated and compared.

### 2.5. ANTS/DPX liposome leakage assays

The liposomes contained a fluorescent dye, 8-aminonaphthalene-1,3,6-trisulfonic acid disodium salt (ANTS), and a quencher, *p*-xylene-*bis*-pyridinium bromide (DPX). Upon release of these compounds from liposomes, the increase of fluorescence (λ_ex_ = 350 nm, λ_em_ = 550 nm) was monitored on a QuantaMaster Fluorometer (Photon Technology International, Lawrenceville, NJ, USA) based on procedures of prior studies [14,40-42]. A small aliquot of an appropriately diluted liposome preparation (100 μL) was injected into a magnetically stirred quartz cuvette containing 3 mL of a pH 7.4 buffer (5 mM HEPES and 145 mM NaCl). The total lipid concentration in the cuvette was 0.12 mM unless indicated otherwise. Once the fluorescence was stable, 50 μL 1M acetic acid was injected into the cuvette to decrease the pH and the fluorescence was monitored until it plateaued. The liposomes were then permeated by the detergent C_12_E_8_ (20 μL of 10% solution in water) to achieve exhaustive leakage (100% leakage). Fluorescent intensity was recorded continuously during the experiment and the raw fluorescent intensity data were converted to ASCII format. The ASCII data was then processed to percentage of contents leakage with Windows Excel software as previously described [41].

To study the pH-dependence of the liposome leakage we used a series of buffers with pH 8.4, 7.2, 6.5, 6.0 (5 mM HEPES, 145 mM NaCl) and pH 5.5, 4.9, 4.5, 4.0, 3.6 (50 mM acetic buffer, 145 mM NaCl). After injection of a liposome preparation into a buffer and stabilization of the fluorescence as described above, the detergent was added to completely permeate the liposomes, and the fluorescence intensity was determined as “100% leakage”.

### 2.6. Freeze-fracture electron microscopy of liposomes

Two liposome samples were characterized by freeze-fracture electron microscopy [25-28] at Nano Analytical Laboratory (San Francisco, CA). Sample 1: the **1**/POPC/PEG-ceramide (50/45/5) liposomes (5 mM total lipids) prepared in 5 mM HEPES buffer, pH 7.4. Sample 2: the pH of an aliquot of Sample 1 was adjusted to 5.5 by acetic acid 5 min prior to freeze-fracturing.

The liposome samples were quenched in liquid nitrogen-cooled propane, using the sandwich technique, which reached a cooling rate of 10,000 K/s to prevent ice crystal formation or artifacts from the cryofixation process. The cryo-fixed samples were stored in liquid nitrogen for less than 2 h before fracturing with a JEOL JED-9000 freeze-etching instrument. The exposed fracture planes were shadowed with Pt for 30 s at an angle of 25-35 degrees and then with carbon for 35 s (2 kV/60–70 mA, 1 × 10^−5^ Torr). The replicas thus produced were cleaned with concentrated, fuming HNO_3_ for 24 h followed by agitation with fresh chloroform/methanol (1/1, v/v) at least 5 times. The cleaned replicas were examined with a JEOL 100 CX electron microscope.

### 2.7. Equilibrium microdialysis assay for methotrexate leakage

The leakage of the anticancer drug MTX from the liposomes was measured by equilibrium microdialysis [31] using the Rapid Equilibrium Dialysis Device (Thermo Scientific) according to the manufacturer's recommendations (inserts with membrane, MWCO = 8,000). For samples without detergent, 50 μL of 1 M acetic acid and 20 μL of HEPES buffer (pH 7.4; 5 mM HEPES, 140 mM NaCl) were added to 300 μL of a liposome preparation (3 mM total lipid concentration) to adjust pH to 5.5. In control experiments (no acid, pH 7.4), 70 μL of HEPES buffer was added to the liposome preparation. For samples to be permeated by detergent, 50 μL of 1 M acetic acid and 20 μL of 2% C_12_E_8_ were added to 300 μL of a liposome preparation (3 mM total lipid concentration) to adjust pH to 5.5. The mixture was transferred into a sample chamber of the Rapid Equilibrium Dialysis Device, and HEPES or acetic buffer (500 μL with the same pH as the sample) was added into a buffer chamber. The sample was dialyzed at room temperature for 4 hours. An aliquot of the aqueous media in the buffer chamber was diluted 5-fold and its UV absorbance (A) was measured at 306 nm. The extinction coefficient of MTX was confirmed to be identical at pH 5.5 and pH 7.4. The percentage of leakage of MTX (Leakage %) due to the drop of pH from 7.4 to 5.5 was calculated according to the formula, Leakage % = (A_5.5_ – A_7.4_)/(A_d_ – A_7.4_) × 100%, where A was the UV absorbance after the sample had been dialyzed at the corresponding pH. A_d_ was the UV absorbance after the liposomes had been permeated by C_12_E_8_ and then dialyzed at pH 5.5.

### 2.8. MTS cytotoxicity assay on HeLa cells

To test the anti-proliferation effect of the MTX-loaded liposomes we used CellTiter 96 AQ_ueous_ One Solution cell proliferation assay (MTS reduction) [43]. The folate-deficient medium (FDMEM) was used, because MTX is a competitive inhibitor of dihydrofolate reductase [29,30]. HeLa (human cervical cancer) cells were continuously cultured in FDMEM containing 10% heat-inactivated fetal bovine serum, penicillin (50 I.U./mL), streptomycin (50 μg/mL), 2% NaHCO_3_, 2 mM L-glutamine and non-essential amino acids in a humidified atmosphere (T75 flasks, 37 °C, 5% CO_2_). At 80–90% confluency, the cells were detached by trypsin, diluted with FDMEM, and seeded in 24-well plates (100 μL suspended cells at 5 × 10^5^ cells/mL plus 400 μL FDMEM for each well). The cells were cultured for another 24 h to reach 70–75% confluency. FDMEM in the well plates were replaced with serum-free FDMEM (400 μL/well), and into each well was added 100 μL of a liposome formulation appropriately diluted by serum-free FDMEM, followed by gentle agitation. The final total concentration of MTX was 1 μM. After 24 h of incubation, the medium was aspirated and the cells were washed twice with phosphate buffered saline (PBS). The cells were then supplemented with FDMEM and cultured for another 6 h.

The cell viability agent MTS [3-(4,5-dimethylthiazol-2-yl)-5-(3-carboxymethoxyphenyl)-2-(4-sulfophenyl)-2H-tetrazolium, inner salt, 100 μL/well] was added and the cells were incubated for 2 h at 37 °C, 5% CO_2_. To stop the redox reaction, sodium dodecylsulfate (10%, 150 μL/well) was added, and the absorbance of the media at 490 nm (A_490_) was measured with an automated plate reader (Tristar, Brethold Technology, Austria). All data were normalized by the A_490_ of HeLa cells treated with serum-free FDMEM into percentage of viability and reported as mean ± SD of at least eight replicate wells.

## 3. Results and Discussion

### 3.1. pH-Triggered conformational flip of lipid **1**

The key concept of our liposome design is the incorporation into lipid molecules of a pH-responsive switch that performs a drastic conformational flip upon protonation, thus disturbing the lipid bilayer and inducing contents release.

We explored the conformational chair-flip of lipid **1** (Figure 1) by ^1^H NMR (600 MHz). The vicinal coupling constants ^3^*J*_HH_ between several protons attached to the cyclohexane moiety of **1** are strongly conformation-dependent, which allows an assignment of a predominant conformation and an estimation of a position of conformational equilibrium. The conformer populations (*n**_A_**, n**_B_***) in the fast equilibrium [**1A + 1A**H^+^] **⇆**[**1B + 1B**H^+^] (Figure 1) were calculated as described before for (amino)cyclohexanol derivatives [14,32-36] from an averaged signal width (*W* = Σ*J*_HH_) measured as the distance between terminal peaks of a multiplet in ^1^H NMR: ***W_observed_* = *W_A_*·*n_A_* + *W_B_*·*n_B_***. We used mainly the signal of H4 (geminal to the hydroxy group, Figure 1), which was usually better resolved and located in a region apart from other signals. The limiting parameter *W_B_* was assumed to equal *W_BH+_*, *i.e.*, the value observed in presence of excess acid (Σ*J*_HH_ = 10.55 + 10.55 + 4.4 = 25.5 Hz), and the parameter *W_A_* was estimated as 9.0 Hz [14,32-36] from reported data for the related conformationally biased aminocyclohexanols. The estimated share of the conformer **1A** H^+^ includes both the non-protonated form **1A** and the protonated form **1A·**H^+^ (the latter being apparently the minor one due to conversion into **1B·**H^+^). The share of the conformer **1B** (*n_B_*) includes both the protonated form **1B·**H^+^ (apparently the major one in access acid) and the non-protonated form **1B**. The coupling constants for other protons of the cycle, e.g. H5, were used when possible to confirm the conformational assignment.

**Figure 2.**
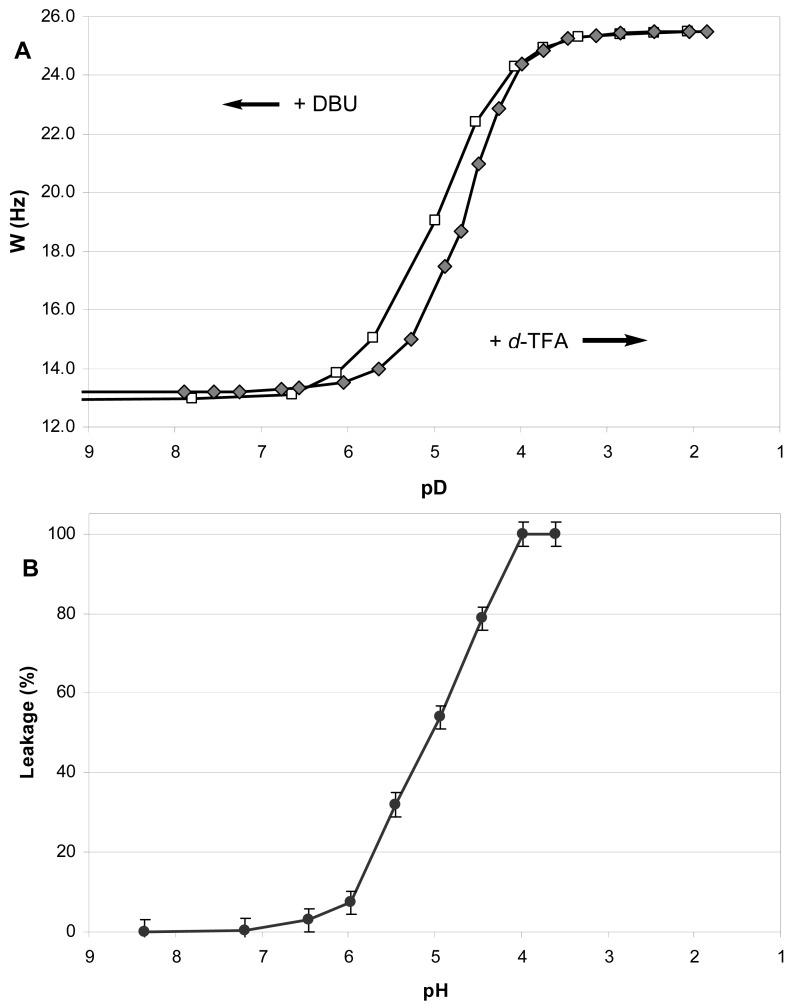
(A) Change of the signal width (W = Σ*J*_HH_) for the proton H4 in ^1^H NMR due to the conformational switch of flipid **1** in CD_3_OD solution caused by titration with *d*-TFA (◆) and then by backward titration of the resulting acidic solution with DBU (□). (B) pH-Dependence of the ANTS/DPX leakage from liposomes with 50 mol% of flipid **1**.

During the incremental addition of acid, the signal width of H4 in **1** increased from the original 13.2 Hz at apparent pD 7.9 to eventually 25.5 Hz at pD 1.85 (Figure 2A), indicating a strong protonation-induced shift of the conformational equilibrium from **1A** (∼75% in original solution) to **1B**H^+^ (∼100% in excess acid). The H5 signal of **1** (*W* = 13.8 Hz at pH 7.9) also widened upon acidification to *W* = 26.5 Hz, and migrated from δ 2.23 downfield to 63.26 because of the deshielding effect of the positive charge at the geminal nitrogen. On the contrary, the multiplet H4 shifted *upfield* from δ3.99 to δ3.90 despite of the deshielding effect of the neighboring morpholinium group. Because *axial* protons typically give a signal at higher field than the otherwise equivalent *equatorial* protons, this observation confirms an *equatorial*-to-*axial* change of H4, and thus the *axial*-to-*equatorial* change of the hydroxy group. Further, the originally well-resolved signals of H1 (δ2.98; *W* = 22.5 Hz) and H2 (δ3.08; *W* = 23.3 Hz) in **1** became narrow and unresolved, and both shifted downfield to δ 3.3-3.4 upon the drop of pD. This attests to an *axial*-to-*equatorial* switch of H1 and H2, and thus to the *equatorial*-to-*axial* switch of the ester groups with lipid tails (Figure 1).

The NMR titration curve (Figure 2A) revealed an interesting peculiarity: the change in a conformer population did not occur gradually upon addition of acid, but took place rapidly between pD 5.5 and 4.0. We observed previously such a rapid conformational switch within a certain range of acidity for the simple model *trans*-2-aminocyclohexanols [35] and in the preliminary study of lipid **1** [14].

In order to confirm that the observed pH-triggered conformational flip is reversible and nondestructive, we back-titrated the resulting acidic solution of lipid **1** in CD_3_OD with a strong base DBU (Figure 2A). As the apparent pD increased above 4.0, an opposite conformational switch occurred rapidly. Upon addition of excess base, the conformational equilibrium returned finally to the predominance of the conformer **1A** (Figure 1). The base-titration curve is similar to the acid-titration curve but its central part is somewhat shifted to the left of the pD-axis. Thus the backward change of equilibrium happens over a wider range of pD - from 4.0 to 6.0, meaning that it takes a somewhat more basic environment than the original one to achieve the original ratio of conformers. Along the W-axis, the backward curve is above the forward curve indicating a higher population of the form **1B**H^+^ (up to 16% additionally) at the same pD values during the base-titration as compared to the acid-titration. Such a “hysteresis” may result from an increased polarity of solution due to accumulation of the ionic DBU·H^+^ CF_3_COO^−^ when TFA is neutralized by DBU in the course of base-titration.

The ^1^H NMR spectrum of the fully back-titrated **1** did not show a significant difference from the original one before the titrations (except for additional signals of DBU and its salt), confirming that the lipid survived this pH-induced forward-backward conformational flip.

Thus the ^1^H NMR titration demonstrated the ability of lipid **1** to perform a dramatic conformational flip in response to variation of pH (pD), and such a flip would substantially affect the packing of liposome bilayers (see the further discussion). Therefore, it is justified to underscore this unusual feature of a quick and reversible alteration of lipid properties by the term *‘flipid’*.

Contrary to flipid **1**, the lipids **2** and **3** (Figure 1) were designed not to perform a pH-induced conformational flip: the previous studies confirmed that the formation of intramolecular hydrogen bond in **2** or **3** upon protonation does not require an interconversion of the originally predominant (∼95% by NMR) conformation **A** [14].

### 3.2. Preparation and characterization of liposomes

PEGylated liposomes that encapsulated either ANTS/DPX or methotrexate were prepared by the freeze-thawing method [37]. Starting with any particular lipid composition, this method yielded liposomes of reproducible colloidal properties and thus allowed studies on the relationship between the lipid composition of the liposomes and their pH-triggered release of contents. ^1^H NMR analyses of the chloroform extract of the **1**/POPC/PEG-ceramide liposomes also confirmed that the molar ratio of the lipid components remained constant after the liposome preparation.

Liposome formulations containing the amphiphile **1**, different neutral phospholipids [phosphatidyl choline (PC) or phosphatidyl ethanolamine (PE)], and different PEG-lipid conjugates (PEG-ceramide or PEG-DPPE) in various mole percentages were prepared at pH 7.4, and their colloidal properties were characterized (Table 1).

After freeze-thawing and extrusion through 200 nm polycarbonate membranes, all the liposomes showed a homogeneous hydrodynamic diameter below 200 nm (polydispersity index < 0.2) except for those whose membranes contained the highest mole percentage of **1** (90 mol%). This observation indicates that **1** by itself is not satisfactory for lipid bilayer formation at pH 7.4 unless other types of lipids are incorporated at sufficient mole percentage.

Knowledge of liposomal ζ-potential can help to predict the fate of liposomes *in vivo* [44,45]. All liposome preparations showed near-zero ζ-potential values at pH 7.4 (Table 1) as expected from the neutrally charged lipids used for liposome construction, which suggest a potentially low toxicity [45].

After storage at 4 °C for about 18 months, the best liposome formulations - **1**/POPC/PEG-ceramide and **1**/POPC/PEG-DPPE (all ratios, except 5/90/5) – did not show noticeable changes in hydrodynamic diameter or polydispersity index, and kept a near-zero ζ-potential. The **1**/DPPC/PEG-ceramide (50/45/5) liposomes revealed some aggregation after 2.5 months storage. The liposomes comprising lipids **2** and **3** showed significant precipitation after 2–3 weeks storage.

The **1**/POPC/PEG-ceramide (50/45/5) liposomes containing ANTS/DPX were also stable in serum. After incubation with serum (1/3 = v/v) at 37 °C for 48 h, they leaked only 6% of the total content.

### 3.3. pH-Triggered leakage of liposomes as a result of conformational flip

The amphiphile **1** was designed to change its predominant conformation in acidic environment thus causing disturbance in the structure of a lipid bilayer, which in turn allows leakage of liposome content. The ^1^H NMR studies demonstrated that flipid **1** does indeed change its conformation when acidity increases, and this flip occurs in *d_4_*-methanol solution between apparent pD 5.5 and 4.0 for the acidic titration and between pD 4.0 and 6.0 for the basic back-titration (section 3.1). To further validate the concept, we studied the pH-dependence of the leakage from liposomes containing flipid **1** in their bilayer.

We measured the leakage of ANTS/DPX from the **1**/POPC/PEG-ceramide liposomes (molar ratio 50:45:5) after injecting a small aliquot of the liposome preparation into buffer solutions with pH varying from 8.4 to 3.6 (Figure 2B). After stabilization of fluorescence, its value was measured and normalized by subtraction of the fluorescence at pH 8.4 (0% leakage at Figure 2B). The percent leakage was calculated by comparing to the maximal value obtained after addition of detergent (100% leakage at Figure 2B). We obtained practically the same values of the percent leakage in three sets of measurements, one of which was done for the liposome preparation stored at 4 °C for 7 months.

As the initial horizontal part of the titration curve shows, the liposome leakage does not occur in basic or neutral medium. However, the increase of acidity brings about a dramatic jump of leakage between pH 6.0 and 4.0. The resulting diagram is strikingly similar to the NMR-titration curve for the conformational flip of **1** (Figure 2A), especially to the backward titration of the acidic solution of **1** in CD_3_OD with DBU. One may hypothesize that the methanol solution with ionic species accumulated in the course of the back-titration is a better model for the polar environment of liposomes in the aqueous buffer solutions. We consider the similarity between the titration curves (A) and (B) (Figure 2) as an evidence of the intrinsic dependence of the pH-induced liposome leakage on the pH-triggered conformational flip of the amphiphile **1**.

Another important evidence for the role of conformational flip in the liposome leakage was obtained from the comparison of lipids **1** and **3**. Lipid **3** differs from flipid **1** only in the configuration of one lipid tail substituent, and for this reason it is unable to noticeably change its conformation after protonation (Figure 1) [14]. Thus the results for the control **3**/POPC/PEG-ceramide liposomes (Table 1) allowed observation of all possible effects of the protonation of morpholine group on the liposome permeability (change of headgroup radius, change of hydrogen bondings, *etc.*), except for the effects caused by the change of conformation. Therefore, the much larger and faster leakage of **1**/POPC/PEG-ceramide liposomes than of **3**/POPC/PEG-ceramide liposomes at pH 5.1 (Table 1) can be attributed to the pH-triggered conformational change of the lipid tails in **1**. As expected, the control liposomes containing neither **1**, nor **3** (last three entries in Table 1) were not responsive to lowered pH.

To highlight the key role of the conformational flip of the cyclohexane ring in the pH-triggered liposome leakage, we would like to introduce the term *‘fliposomes’* for the liposomes comprising lipids equipped with such a pH-sensitive conformational switch.

### 3.4. The effect of the mole percentage of **1** on the pH-sensitivity of fliposomes

We performed most liposome leakage assays using the standardized procedures of prior studies [14,40-42], in which a small aliquot of liposome preparation was injected into pH 7.4 buffer, then acetic acid was added to decrease the pH, and the fluorescence was monitored until it plateaued. The exhaustive leakage (100%) was estimated after addition of detergent.

**Figure 3.**
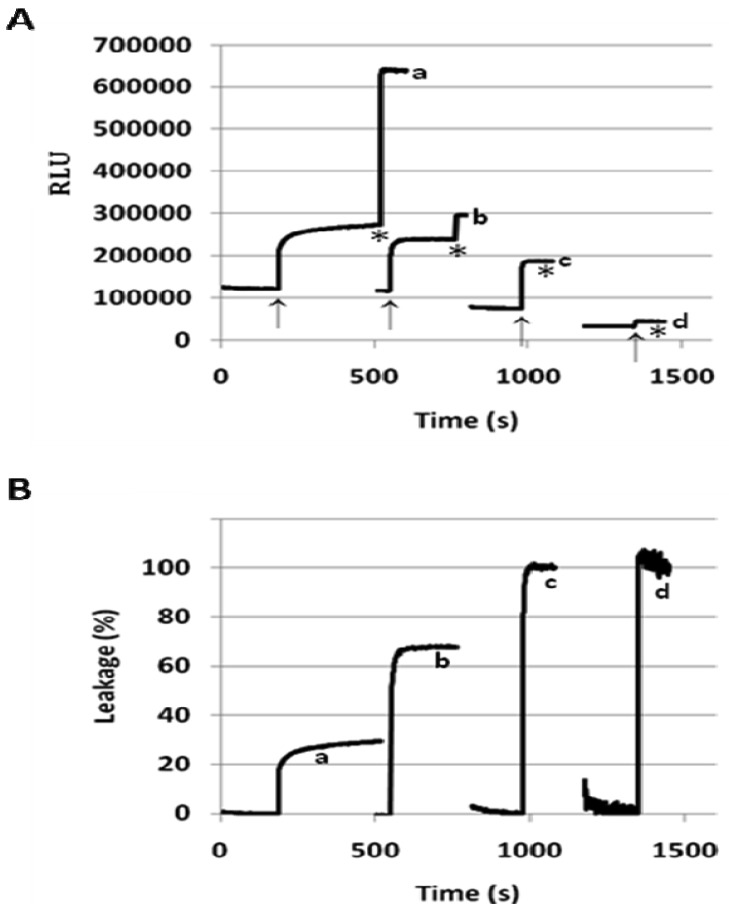
Effect of the mole percentage of **1** on the pH-triggered release of ANTS from **1**/POPC/PEG-ceramide fliposomes. The release is presented: (**A**) in relative light units (RLU) and (**B**) in percent of leakage. The molar ratio **1**/POPC/PEG-ceramide: a, 25/70/5; b, 50/45/5; c, 75/20/5; d, 90/5/5. Arrows indicate addition of acid to lower pH from 7.4 to 5.1; asterisks indicate addition of C_12_E_8_ for fliposomes permeation. The *x*-axes are in relative time scales to fit all the traces into the figure.

When incorporated into the lipid membranes at 25 mol%, flipid **1** induced substantial leakage (23–35%) of a variety of PEGylated liposomes at lowered pH (Table 1), including those consisting of saturated lipids, unsaturated lipids, PEs or PCs, and two different types of PEG-lipid conjugates. Since the liposomes containing POPC showed greater pH-triggered release than those consisting of other phospholipids, such liposomes were selected for further studies. In order to examine whether the pH-sensitivity of the liposomes can be improved by incorporating higher mole percentage of **1** into the liposome membrane, the **1**/POPC/PEG-ceramide liposomes containing 25–90 mol% of **1** were prepared and characterized (Figure 3, Table 1). Upon exposure to pH 5.1, the liposomes containing 25 mol% **1** released about 20% of ANTS instantly (in a few seconds), and up to 30% in approximately 5 min. As the mole percentage of **1** was increased, both the rate and the extent of the release of contents were increased. At 50 mol% of **1**, about 70% of ANTS was released in a few seconds. As the mole percentage of **1** was further increased (75 mol% and 90 mol%), the liposomes released all the contents in even fewer seconds. However, at higher than 50 mol% of **1**, the encapsulation efficiency of the liposomes deteriorated severely as shown by much lower fluorescence of ANTS after its exhaustive release in the presence of the detergent C_12_E_8_. It appears that 50 mol% of **1** is optimal for balancing efficient release from PEGylated, POPC-containing liposomes at pH 5.1 and high encapsulation capacity at pH 7.4.

### 3.5. Effect of PEG-lipid conjugates on the pH-triggered release of fliposomes

**Figure 4.**
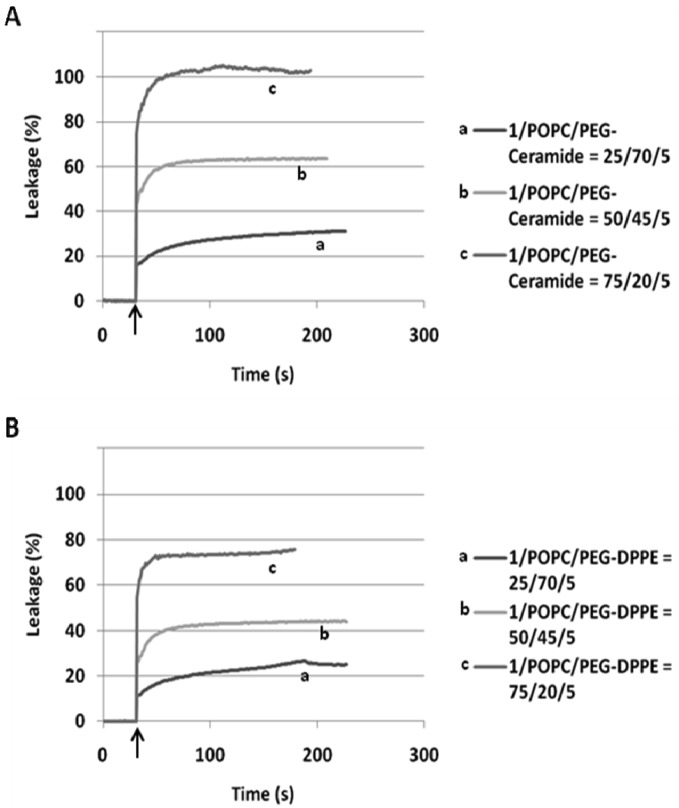
Release of ANTS from fliposomes containing (**A**) **1**/POPC/PEG-ceramide or (**B**) **1**/POPC/PEG-DPPE upon lowering pH from 7.4 to 5.1 (arrows). The *x*-axes are in relative time scales to fit all the traces into the figure.

In our preliminary report on flipid **1** [14], the neutrally charged PEG-lipid conjugate, PEG-ceramide was incorporated into the liposome membrane as a means of grafting the liposome surface with PEG. PEG-ceramide was chosen over PE-PEG conjugates [46–48] to avoid potential ionic interactions between the negatively charged phosphate group of PE-PEG and the positively charged ammonium group of protonated **1**. To further determine whether **1** is capable of triggering liposomes consisting of PE-PEG conjugates, which have been much more widely used in long-circulating drug/gene delivery systems [7,49,50], the **1**/POPC/PEG-DPPE liposomes containing different mole percentages of **1** were constructed and their pH-dependent leakage characterized in comparison with **1**/POPC/PEG-ceramide liposomes (Figure 4). Similar to the **1**/POPC/PEG-ceramide liposomes, the **1**/POPC/PEG-DPPE liposomes quickly released the encapsulated ANTS in response to the lowered pH. The higher the mole percentage of **1** in the liposomes, the higher was the percentage of the leakage. All other conditions being equal, the incorporation of PEG-DPPE in place of PEG-ceramide decreased the percentage of leakage at pH 5.1. For example, approximately 70% of ANTS was released from **1**/POPC/PEG-DPPE liposomes with 75 mol% of **1**, compared to 100% ANTS release from the corresponding **1**/POPC/PEG-ceramide liposomes.

### 3.6. pH-Triggered release from **1**/POPC/PEG-ceramide fliposomes does not rely on inter-liposome bilayer contact

**Figure 5.**
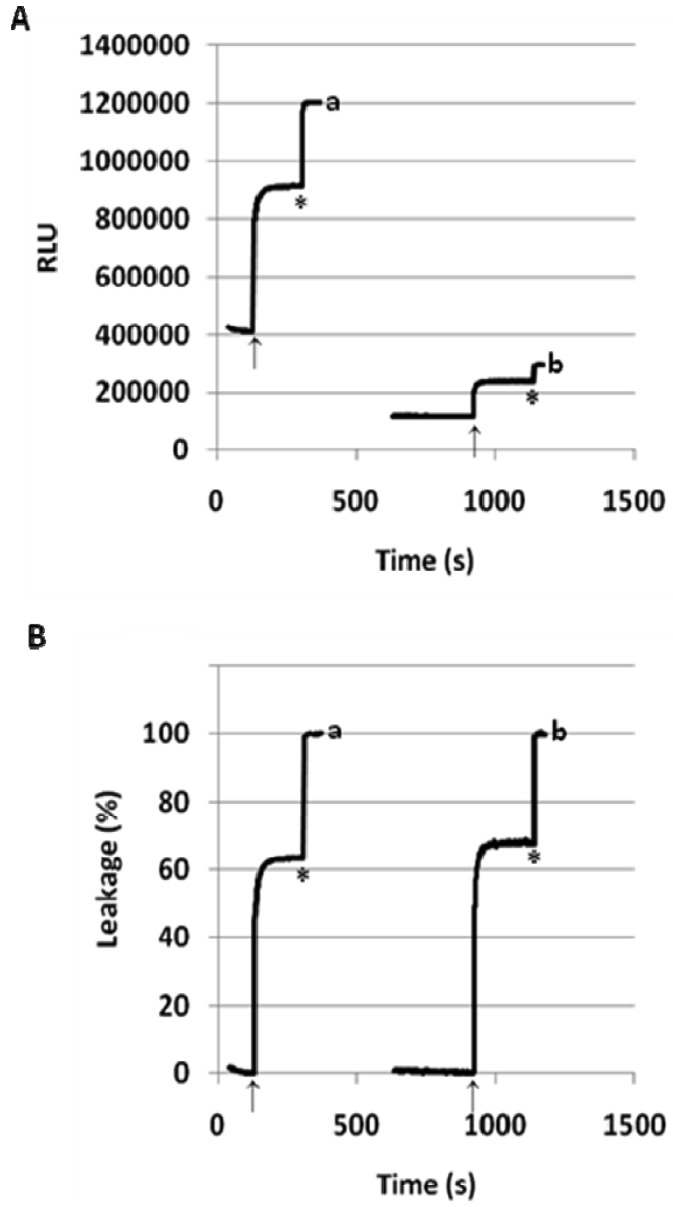
pH-triggered release of ANTS from **1**/POPC/PEG-ceramide fliposome (50/45/5) at (a) 0.12 mM and (b) 0.024 mM total lipid concentrations. Arrows: pH lowered from 7.4 to 5.1; asterisk: fliposome permeated by the detergent C_12_E_8_. The release is expressed: (**A**) in relative light units (RLU), and (**B**) in percent of leakage. The x-axes are in relative time scales to fit all the traces into the figure.

To better understand the mechanism of fliposome leakage, the hydrodynamic diameters of the **1**/POPC/PEG-ceramide fliposomes were measured before and after exposure to pH 5.1 for 1 h (Table 2). Regardless of the mole percentage of **1** or the extent of the leakage, the fliposomes retained their averaged hydrodynamic diameters after being triggered by the lowered pH, indicating that liposome aggregation/fusion is not significantly involved in the triggering process. Furthermore, when the **1**/POPC/PEG-ceramide (50/45/5) liposome was triggered at five times lower concentration (0.024 mM, pH lowered from 7.4 to 5.1), the rate and the extent of ANTS leakage (%) (Figure 5) were similar to those at the default lipid concentration (0.12 mM). This observation confirmed that the liposome content release did not rely on the lipid concentration, and thus did not rely on the inter-liposome bilayer contact, in contrast with most other reported pH-sensitive liposomes [24].

For the purpose of drug delivery, the contact-independent pH-sensitive fliposomes may offer certain advantages. For instance, the fliposomes can retain their pH-sensitivity in the presence of PEG-lipid conjugates, which have been well documented to improve stability in blood circulation and to hinder the inter-liposome bilayer contact. Another potential advantage would be the robust drug release from each fliposome after it reaches certain acidic target sites (e.g., core of solid tumors) where the number of available liposomes may be too low for contact-dependent lipid phase changes.

### 3.7. Morphological changes of the **1**/POPC/PEG-ceramide fliposomes in response to lowered pH

**Figure 6.**
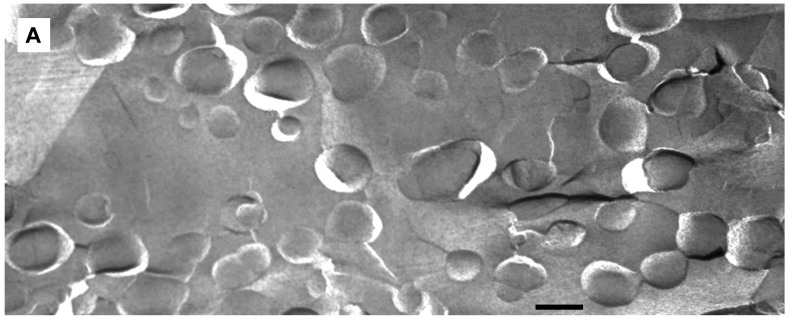

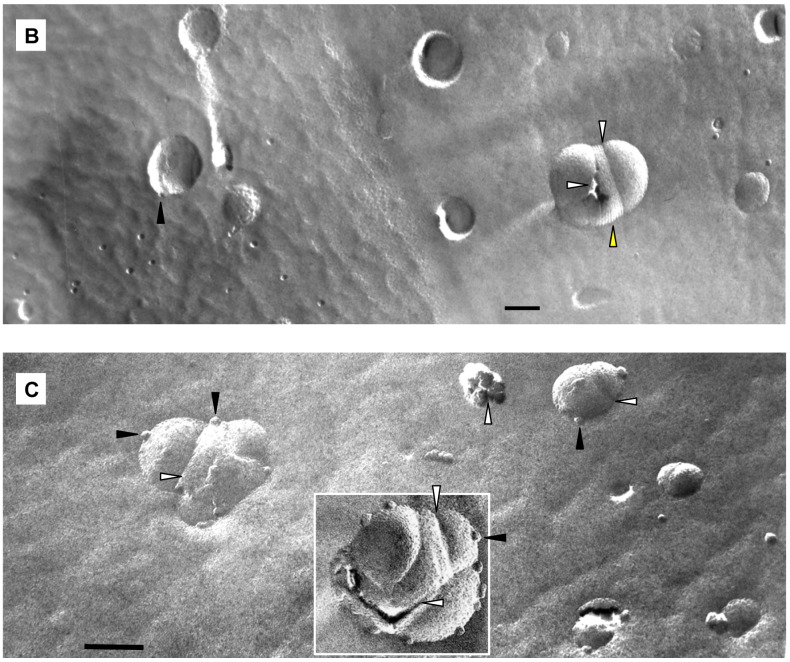
Freeze-fracture electron microscopy of the **1**/POPC/PEG-ceramide fliposome formulation at pH 7.4 (**A**) and 5 minutes after adjusting the pH to 5.5 with diluted acetic acid (**B**, **C**). Examples of division, buds, and stripes are shown with white, black, and yellow arrows respectively. The bars represent 100 nm.

We applied freeze-fracture electron microscopy (FFEM) to gain more insight in the mechanism of triggered release from fliposomes because this powerful technique can not only image structures of lipid colloids at nanometer resolution, but also take snapshots of lipid phase transformation by rapid freezing [25-28]. The unloaded **1**/POPC/PEG-ceramide (50/45/5) fliposome formulation was studied both at pH 7.4 and after exposure to pH 5.5 for 5 min. Electron micrographs were taken from several freeze-fracture preparations of each sample and are presented on Figure 6. At both pH values, the samples contained vesicles. Since these vesicles displayed their shadows both in front of (concave fracture surfaces) and behind their structures (convex fracture surfaces), they are confirmed as liposomes with a shell of lipid bilayer. The diameters of the fliposomes measured on the electron micrographs range from 20 to 230 nm with an average diameter of about 100 nm. They are smaller than determined by photon correlation spectrometry (PCS), possibly for two reasons: one is that FFEM does not reflect the PEGylated outer lipid monolayer, and the other is that the averaged diameter by PCS usually over-represents the lager particles because they scatter light much more efficiently than their smaller counterparts [51].

At pH 7.4 the **1**/POPC/PEG-ceramide fliposome formulation appeared to be composed mostly of unilamellar vesicles together with a few vesicles displaying two bilayers (Figure 6A). At 5 mM total lipid concentration, the fliposomes were close to one another in the micrographs. Occasionally, small aggregates of a few vesicles were also observed. Upon decrease of pH from 7.4 to 5.5, the **1**/POPC/PEG-ceramide fliposomes underwent drastic morphological transformations (Figures 6B, C) that fell roughly into two categories: division and budding. Division frequently took place in larger vesicles, where stripes were observed in the dividing areas. Defects of lipid packing that resembled long and deep cracks were also observed on some of the dividing fliposome membranes. Budding was frequently observed in both smaller and larger vesicles, where small domains of lipids (<9 nm in diameter) protruded out of the fliposome membranes (Figure 6C). It appears that a large number of such lipid domains already pinched off the fliposomes as freely suspended small spherical particles (Figures 6B, C). Some fliposomes showed all of the above-described morphological features (division, stripes, and buds), indicating that the division and the budding events could take place simultaneously on the same fliposome (the complex fliposomes at Figures 6B, C).

**Figure 7.**
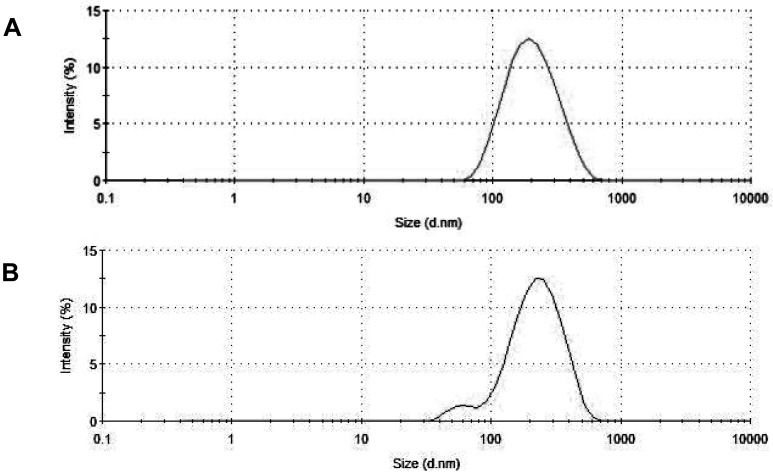
The PCS-estimated size distribution for the unloaded **1**/POPC/PEG-ceramide fliposome formulation at pH 7.4 (**A**) and pH 5.5 (**B**).

The results of size distribution measurements (Figure 7) conform in general to the data of electron microscopy. The PCS-estimated average size of particles changed very little with addition of acid (from ∼180 nm to ∼187 nm). However, the maximum of the distribution curve shifted to somewhat greater sizes (from ∼216 to ∼243), and a broad small-size shoulder emerged at around 60 nm.

### 3.8. pH-Triggered release of the anticancer drug methotrexate from PEGylated fliposomes

Up to date, many reported pH-sensitive lipids managed to trigger liposomes only in cooperation with a high mole percentage of fusogenic PEs [10,11]. In contrast, the flipid **1** successfully destabilized a large variety of liposome membranes at lowered pH, including saturated membranes containing DPPC, unsaturated membranes containing POPC or POPE, membranes containing small (PE) or large (PC) lipid head groups, and membranes grafted with either PEG-ceramide or PEG-DPPE. Such versatility suggests that the aminocyclohexanol-based conformational switches may find applications in diverse delivery systems of therapeutic or diagnostic agents such as saturated, long-circulating liposomes for anticancer drug delivery [52,53], and unsaturated, PE-rich liposomes for gene delivery [54,55] across cellular membranes.

In order to confirm that PEGylated fliposomes are capable of releasing encapsulated drugs in response to lowered pH [56,57], we constructed the **1**/POPC/PEG-ceramide (50/45/5) and the **1**/POPC/PEG-DPPE fliposomes (50/45/5) containing the anionic anticancer drug methotrexate (MTX) [29,30]. After exposure to pH 5.5, both fliposome formulations were subjected to equilibrium micro-dialysis [31] as a more versatile method to quantify drug release regardless of its fluorescence. The percentage of MTX release was determined by measuring the UV absorbance (λ = 306 nm) of the free drug in the solution outside the dialysis bag after the micro-dialysis had reached equilibrium. As shown in Table 3, none of the fliposome formulations released significant amount of MTX after dialysis at pH 7.4 for 4 hours. Upon dialysis at pH 5.5, both fliposome formulations containing flipid **1** released most of the encapsulated MTX. The concentration of MTX generated in the buffer chamber exceeded by far the typical levels of MTX in patients' plasma and serum [58,59]. Interestingly, the percentage of MTX released was higher than that of ANTS from the corresponding fliposome formulations (Table 1), probably due to the much longer duration of the micro-dialysis assay (4 hours *versus* minutes), which would allow the detection of additional slower release of MTX after the initial fast release. For comparison, liposomes containing the diastereomeric amphiphiles **2** or **3** released much less MTX after micro-dialysis at pH 5.5 (Table 3). Such observations confirm that pH-triggered release of contents from fliposomes (ANTS, MTX, *etc.*) results mainly from the conformational change of the lipid tails, which takes place in **1** but not in its diastereomers **2** or **3**.

### 3.9. Anticancer activity of fliposomes against HeLa cells

**Figure 8.**
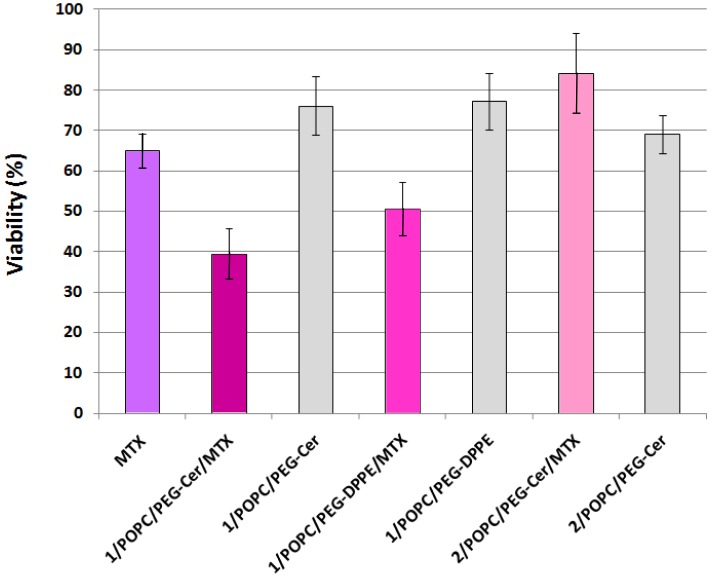
Cytotoxicity of MTX, of the liposome-encapsulated MTX, and of placebo liposomes against HeLa cells. The anticancer cytotoxicity is determined by the MTS assay as the decrease of viability *vs.* untreated cells (100% viability).

Finally, to test whether the fliposomes can serve as a viable drug delivery system, selected liposome formulations encapsulating the anticancer drug MTX were applied to the HeLa cells (human cervical cancer) followed by assessment of the cell viability using the MTS assay [43]. Methotrexate is an active drug in the first-line treatment of the cell carcinoma of the cervix [60,61]. The MTS assay works on the principle that the mitochondrial dehydrogenase of the live HeLa cells reduces the active compound MTS (inner salt) to colored formazan product that can be measured directly at 490 nm. The reduction of MTS by HeLa cells treated with free MTX or with the liposomes was compared to reduction by untreated HeLa cells and reported as relative viability in percentage (Figure 8).

HeLa cells treated with 1 μM (final concentration) of MTX solution retained 65.0 ± 4.3% viability, consistent with prior reports [62-64] on the anticancer activity of the free drug. In comparison, the HeLa cells treated with MTX-loaded fliposomes containing the flipid **1** (**1**/POPC/PEG-ceramide = 50/45/5, and **1**/POPC/ PEG-DPPE = 50/45/5) showed a significantly lower viability (39.4 ± 4.3% and 50.5 ± 6.8%; *p* < 10^−6^ and 10^−3^ by two-tailed Student's T-test, respectively), indicating the superior anticancer cytotoxicity of such liposomal formulations of MTX compared to the free drug at the same dosage.

In contrast, the HeLa cells treated with MTX-loaded liposomes containing the diastereomeric analogue **2** instead of **1** retained most of the viability (84.3 ± 9.9%), thus highlighting the importance of the flipid **1** in enhancing the cytotoxicity of the payload MTX.

As negative controls, the placebo fliposomes (**1**/POPC/PEG-ceramide = 50/45/5, and **1**/POPC/ PEG-DPPE = 50/45/5) without the MTX payload caused much less cytotoxicity (higher viability of 76.1 ± 7.3% and 77.2 ± 7.0%, respectively) than their MTX-loaded counterparts. This result confirmed that the anticancer activity of MTX-loaded fliposomes resulted mostly from the payload drug rather than the vector.

Because the cell culture media were buffered at ∼pH 7.4, the discharge of the **1**/POPC/PEG-ceramide/MTX fliposomes most likely took place in the acidic endosomal compartment of the HeLa cells after their cellular uptake. Because MTX is too hydrophilic to passively diffuse across biomembranes, it is likely that the **1**/POPC/PEG-ceramide/MTX fliposomes not only released MTX in response to the lowered pH in the endosome, but also facilitated the diffusion of the released MTX from the endosome to the cytosol. One possible mechanism of such facilitated diffusion could be a destabilization of the endosomal membranes by the micelles generated from the destruction of fliposomes.

Thus in the HeLa cell culture, the **1**/POPC/PEG-ceramide fliposomes encapsulating MTX demonstrated the highest cytotoxicity. Comparisons with the negative controls (**2**/POPC/PEG-ceramide/MTX and empty **1**/POPC/PEG-ceramide liposomes) confirmed that most of the cytotoxicity resulted from the enhancement of the MTX release by the conformational flip of compound **1**.

### 3.10. Plausible mechanism of the pH-triggered cargo release by fliposomes

Many reported pH-sensitive liposomes are rich in phosphatidylethanolamine (PE) and require inter-liposome bilayer contact in order to respond to the decrease of pH [24,40,41]. In contrast, the lack of aggregation after pH-triggering (Table 2), as well as the concentration-independent release of ANTS (Figure 5), indicates that the **1**/POPC/PEG-ceramide fliposomes do not rely on the inter-liposome contact to undergo pH-sensitive leakage. The fliposomes are also different from most of the reported pH-sensitive liposomes in their extraordinarily fast response: a sufficient decrease of pH releases the fliposome cargo within seconds (Figures 3–5), compared to minutes or even hours that were required for most of the reported pH-sensitive liposomes [24,40,41]. Only some pH-sensitive polymers (e.g., EALA peptide) have been reported to start liposome leakage at a similar rate [13].

Based on the NMR studies on the pH-triggered conformational flip of **1**, the fluorometric and UV-studies on the pH-induced leakage of fliposomes, FFEM images of the morphological changes of **1**/POPC/PEG-ceramide fliposomes (division and budding), and the prior reports on lipid phase transitions, we propose the following unique mechanism of the pH-triggered content release from fliposomes: conformational shortening of the flipid **1** followed by lipid phase separation.

**Figure 9.**
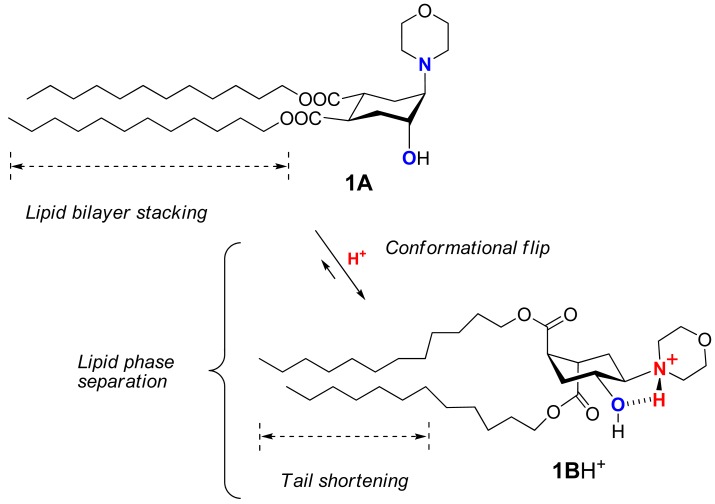
Protonation-induced conformational shortening of flipid **1** causes a quick perturbation of the lipid bilayer, lipid phase separation, and a fast fliposome leakage.

As the flipid **1** is protonated at lowered pH, its headgroup assumes a positive charge and hence a larger hydrodynamic size due to higher hydration. The protonation also generates a strong intramolecular hydrogen bond between the morpholine amine and the neighboring hydroxyl group, which flips the chair conformation of the cyclohexane ring and switches its two remote ester groups from the equatorial to the axial positions, thereby increasing the spatial separation of the two dodecyl groups of **1** (Figure 9). Such spatial separation would be more at the proximal end of the hydrocarbon chains than at their distal end, where conformational freedom and hydrophobic interactions would allow the chains to partially fold back and re-pack in the lipid bilayer. Such curved conformation of the two dodecyl groups (Figure 9) would decrease the packable length of the hydrophobic moiety of **1** in the fliposome membrane, which we refer to as “conformational shortening”.

Such “conformational shortening” of the hydrophobic moiety of **1**, which constitutes in most experiments 50 mol% of the whole lipid composition, could directly perturb the lipid bilayer to cause the fliposome leakage. More likely, the conformational shortening would induce phase separation of the fliposome membrane, e.g. into thinner domains that are rich in **1** and thicker domains that are rich in POPC molecules, which carry a longer hydrophobic moiety. The encapsulated contents of the fliposomes would then quickly leak through defects between the domains. The domains of monolayers that are rich in **1** would subsequently bud from the fliposome membranes as small micelles [65,66], as observed in FFEM. Conversely, the remaining of the fliposome would carry minimal charges but retain a much larger size. Such larger particles would scatter photons much more strongly than the small micelles and therefore dominate the readings of photon correlation spectrometry [51] to show a relatively stable apparent size of the fliposomes after adjusting the pH from 7.4 to 5.1. The formation of micelles from larger **1**/POPC/PEG-ceramide fliposomes would be slower because their outer lipid monolayer carries less positive curvature than the smaller fliposomes. This allows the small domains that are rich in **1** to merge into larger lipid phases such as stripes, which would eventually disperse as micelles to leave defects on the fliposome surface. The kinetically competitive nature of the budding and the processes of the lipid phase separation would explain why both events could be observed in the same fliposome.

Although a lipid translocation (flip-flop) was involved in many reported pH-sensitive lipid phase changes [66-68], it is unlikely to significantly contribute to the pH-triggered release from the fliposomes because of its much slower kinetics (hours for flip-flop [68] *versus* seconds for fliposome leakage).

## 4. Conclusions

By incorporation of the conformationally switchable *trans*-2-morpholinocyclohexanol-based amphiphile (flipid) into lipid compositions, we designed pH-triggerable liposomes (fliposomes) with extraordinary characteristics: high stability in storage and in serum *in vitro* combined with instant release of their cargo in response to a weakly acidic medium. The acid-induced release from fliposomes can be modulated by the mole percentage of the flipid, and is independent of inter-liposome contact. Based on fluorometric, NMR and electron microscopy studies, a mechanism of pH-sensitivity has been proposed that starts with an acid-triggered conformational flip and is followed by a set of membrane perturbations, which cause the leakage. Anticancer activities of selected methotrexate formulations in HeLa cells demonstrate that fliposomes can serve as viable drug delivery systems.

## Figures and Tables

**Table 1.** Size, ζ-potential and pH-triggered leakage of PEGylated liposomes.

**Lipid Formulations**	**Molar Ratio**	**Upon Preparation**			**After Storage**		**Leakage at pH 5.1^4^ (%)**
		**D^1^ (nm)**	**P.I.^2^**	**ζ 3(mV)**	**D^1^ (nm)**	**P.I.^2^**	
**1**/DPPC/PEG-ceramide	25/70/5	138.9	0.08	−7.1	139.7^5^	0.09^5^	27
**1**/DPPC/PEG-ceramide	50/45/5	141.2	0.18	−3.4	204.1^5^	0.29^5^	42
**1**/POPC/PEG-ceramide	25/70/5	146.3	0.13	−6.5	141.5^6^	0.09^6^	35
**1**/POPC/PEG-ceramide	50/45/5	162.4	0.069	−2.8	154.5^6^	0.01^6^	67
**1**/POPC/PEG-ceramide	75/20/5	158.5	0.138	N.D.	164.9^6^	0.18^6^	95
**1**/POPC/PEG-ceramide	90/5/5	233.4	0.41	+4.1	229.5^5^	0.29^5^	100
**1**/POPC/PEG-DPPE	25/70/5	167.8	0.01	−4.9	158.8^6^	0.03^6^	27
**1**/POPC/PEG-DPPE	50/45/5	160.1	0.04	−2.2	172.7^6^	0.14^6^	46
**1**/POPC/PEG-DPPE	75/20/5	184.6	0.18	−4.6	184.5^6^	0.14^6^	75
**1**/POPC/PEG-DPPE	90/5/5	233.1	0.11	−3.7	N.D.	N.D.	100
**1**/POPE/PEG-ceramide	25/70/5	145.1	0.12	+3.5	139.15	0.23^5^	23
**1**/POPE/PEG-ceramide	50/45/5	175.9	0.01	+6.4	183.5^7^	0.06^7^	47
**2**/POPC/PEG-ceramide	25/70/5	197.3	0.14	−3.1	Prp. 8	N.D.	28^9^
**3**/POPC/PEG-ceramide	25/70/5	282.6	0.34	−11.9	N.D.	N.D.	5^9^
**3**/POPC/PEG-ceramide	50/45/5	332.0	0.42	−4.7	N.D.	N.D.	10^9^
DPPC/PEG-ceramide	95/5	151.0	0.07	−7.2	149.3^7^	0.08^7^	< 3
POPC/PEG-ceramide	95/5	146.8	0.17	+3.7	146.3^7^	0.05^7^	< 3
POPE/PEG-ceramide	95/5	194.2	0.16	+1.4	178.5^7^	0.11^7^	< 3

All the listed liposomes contain the fluorophores ANTS/DPX. Six control liposomes are also listed at the bottom of the table for comparison.

1 Hydrodynamic diameter, representation of two to three separate liposome preparations with less than 10 nm variation;

2 PI = polydispersity index;

3 ζ-potential, representation of two to three separate liposome preparations with less than 3 mV variation;

4 Percentage of ANTS released from the liposome after lowering pH from 7.4 to 5.1, average of two to three separate liposome preparations with less than 5% variation;

5 After storage at pH 7.4, 4 °C for 70 days;

6 After storage at pH 7.4, 4 °C for 1.5 years;

7 After storage at pH 7.4, 4 °C for 50 days;

8 Precipitation observed at the bottom of the tube;

9 Percentage of the ANTS release after lowering pH from 7.4 to 5.2–5.3.

**Table 2.** Hydrodynamic diameter of **1**/POPC/PEG-ceramide fliposomes at pH 7.4 and after exposure to pH 5.1 for 1 h.

**Lipid Composition (molar ratio)**	**pH 7.4**		**1 h at p H 5.1**	
	**Diameter (nm)**	**P.I.**	**Diameter (nm)**	**P.I.**
**1**/POPC/PEG-ceramide (25/70/5)	146.3	0.130	144.5	0.135
**1**/POPC/PEG-ceramide (50/45/5)	162.4	0.069	161.2	0.132
**1**/POPC/PEG-ceramide (75/20/5)	158.5	0.138	152.1	0.190
POPC/PEG-ceramide (95/5)	146.8	0.170	142.8	0.241

All the liposomes listed in the table contained the fluorophore ANTS/DPX; P.I.: polydispersity index.

**Table 3.** pH-Triggered release of methotrexate from PEGylated liposomes.

	**MTX concentration (μM)**				**Leakag (%)**
	**pH 7.4**	**pH 7.4 + C_12_E_8_**	**pH 5.5**	**pH 5.5 + C_12_E_8_**	
**1**/POPC/ PEG-ceramide (50/45/5)	61	601	560	642	86
**1**/POPC/ PEG-DPPE (50/45/5)	42	461	380	469	79
**2**/POPC/ PEG-ceramide (50/45/5)	45	382	85	411	11
**3**/POPC/ PEG-ceramide (50/45/5)	42	457	100	427	15

The pH of the liposome samples (300 μL) was adjusted with acetic acid (0-50 μL) with or without the detergent C_12_E_8_, followed by micro-dialysis. The **2**/POPC/PEG-ceramide (50/45/5) and **3**/POPC/PEG-ceramide (50/45/5) liposomes were characterized in parallel as conformationally non-switchable controls. The optical density of the buffer in the outer chamber (500 μL) was used to calculate the MTX concentration and the percentage of leakage (Leakage%).

## Abbreviations

ANTS: 8-aminonaphthalene-1,3,6-trisulfonic acid disodium salt
detergent C_12_E_8_: octaethyleneglycol monododecyl ether
DBU: 1,8-diazabicyclo[5.4.0]undec-7-ene
DPPC: 1,2-dipalmitoyl-*sn*-glycero-3-phosphocholine
DPX: *p*-xylene-bis-pyridiniumbromide
FDMEM: folate-deficient medium
FFEM: freeze-fracture electron microscopy
HEPES: 2-[4-(2-hydroxyethyl)piperazin-1-yl]-ethanesulfonic acid
MTX: methotrexate
PC: phosphatidyl choline
PCS: photon correlation spectrometry
PE: phosphatidylethanolamine
PEG: polyethyleneglycol
PEG-ceramide: N-palmitoyl-sphingosine-1-(succinyl[methoxy(polyethylene glycol)2000])
PEG-DPPE: 1,2-dipalmitoyl-*sn*-glycero-3-phosphoethanolamine-N-[methoxy(polyethylene glycol)-2000]
POPC: 1-palmitoyl-2-oleoyl-*sn*-glycero-3-phosphocholine
POPE: 1-palmitoyl-2-oleoyl-*sn*-glycero-3-phosphoethanolamine
TFA: trifluoroacetic acid

## Supplementary Data

Supplementary data related to the synthesis of compounds **1**–**3** can be found online at doi: 10.3390/pharmaceutics3030379.

Supplementary File 1 PDF-Document (PDF, 95 KB)
