# Supplementary material for: Fliposomes: pH-Sensitive Liposomes Containing a trans-2-morpholinocyclohexanol-Based Lipid That Performs a Conformational Flip and Triggers an Instant Cargo Release in Acidic Medium
Source: Pharmaceutics. 2011 Jul 11;3(3):379–405. doi: 10.3390/pharmaceutics3030379 (PMC3857072; doi:10.3390/pharmaceutics3030379)
Supplement: Supplementary File 1 — PDF-Document (PDF, 95 KB) [file pharmaceutics-03-00379-s001.pdf]

Supplementary Data—Syntheses

The *trans*-2-morpholinocyclohexanol-based amphiphile **1** was prepared as previously described [1]. 2-Morpholinocyclohexanols **2** and **3** with *cis*-configuration of the ester groups were prepared as described below.

Column chromatography was performed on silica gel (Sorbent Technologies, 40–75 μm). The reactions were monitored by TLC (silica gel, 8 × 2 cm plates with UV-indicator (254 nm), Analtech Inc.). ^{1}H NMR and ^{13}C NMR spectra were acquired on Varian Mercury (300 MHz) and JEOL ECA-600 (600 MHz) NMR-spectrometers. Exact mass measurements were performed on JEOL LC-Mate double-focusing mass spectrometer (Peabody, MA, USA) equipped with an electrospray ionization source at a resolving power of 5000 with polyethyleneglycol as an internal reference. The MS spectra were obtained on Varian 1200LC triple quadrupole mass spectrometer (Walnut Creek, CA, USA) with an electrospray ionization source in positive mode. Elemental analyses were carried out by Micro-Mass Facility, UC Berkeley. All solvents were purified by conventional techniques prior to use.

Figure 1S. Synthesis of *trans*-2-morpholinocyclohexanols **2** and **3** with *cis*-configuration of lipid tails.

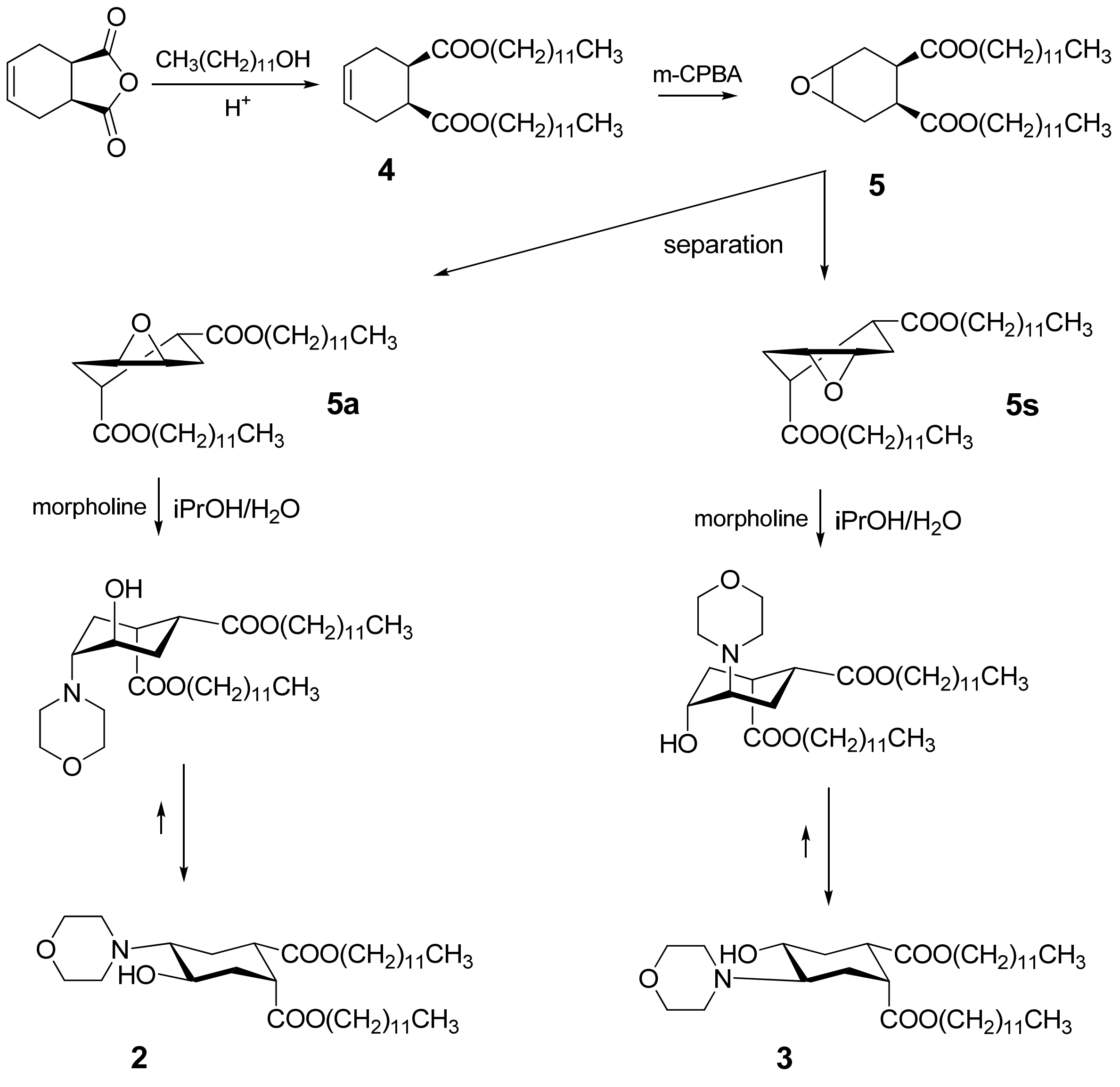

Didodecyl 4-cyclohexene-cis-1,2-dicarboxylate **(4)**. *cis*-Tetrahydrophthalic anhydride (10 g, 66 mmol), 1-dodecanol (32 mL, 26.7 g, 143 mmol), and *p*-toluensulfonic acid (0.5 g) were refluxed 12 h in 200 mL of toluene. Toluene was removed on a rotary evaporator, and the residue was purified by silica gel column chromatography (hexane:EtOAc, 4:1) to yield colorless oil: yield 30.5 g (91%). ^{1}H NMR (300 MHz, $CDCl_3$): 5.65 (m, 2H, H4+H5), 4.05 (t, J = 6.7 Hz, 4H, OCH_2, dodecyl), 3.01 (br. t, J = 5.6 Hz, 2H, H1+H2), 2.54 (m, 2H, H3+H6), 2.34 (m, 2H, H3+H6), 1.58 (br. quin, J = 6.8 Hz, 4H, CH_2, dodecyl), 1.22-1.33 (m, 36H, CH_2, dodecyl), 0.86 (t, J = 6.8 Hz; 6H, CH_3). ^{13}C NMR (75 MHz, $CDCl_3$): 173.24, 125.18, 64.74, 39.82, 31.89, 29.63, 29.61, 29.56, 29.52, 29.32, 29.24, 28.58, 25.91, 25.89, 22.65, 14.04.

*Didodecyl 7-oxabicyclo[4.1.0]heptane-cis-3,4-dicarboxylates (**5a**) and (**5s**)*

Diester **4** (10 g, 19.7 mmol) was epoxidized as previously described for the synthesis of **1** [1]. Two products—*anti*-epoxide **5a** and *syn*-epoxide **5s** (3:1 by ^{1}H NMR)—were separated by silica gel column chromatography (hexane:EtOAc, 5:1).

Epoxide **5a**. yield 4.3 g (42%). ^{1}H NMR (300 MHz, $CDCl_3$): 4.06 (t, J = 6.7 Hz, 4H, OCH_2, dodecyl), 3.23 (br. s, 2H, H1+H6), 2.90 (br. t, J = 5.1 Hz, 2H, H3+H4), 2.16–2.32 (m, 4H, H2+H5), 1.60 (br. quin, J = 6.7 Hz, 4H, CH_2, dodecyl), 1.19–1.38 (m, 36H, CH_2, dodecyl), 0.88 (t, J = 6.6 Hz, 6H, CH_3). ^{13}C NMR (75 MHz, $CDCl_3$): 172.99, 65.03, 51.61, 37.75, 31.90, 29.64, 29.62, 29.58, 29.53, 29.33, 29.25, 28.56, 25.92, 24.89, 22.66, 14.06. HRMS: $C_{32}H_{58}O_5$ requires *m/z* $[M+Na]^+$ 545.4177, found 545.4160.

Epoxide **5s**. yield 2.5 g (24%). ^{1}H NMR (300 MHz, $CDCl_3$): 4.07 (t, J = 6.7 Hz, 4H, OCH_2, dodecyl), 3.16 (br. s, 2H, H1+H6), 2.64–2.76 (m, 4H, H2-H5), 2.07–2.17 (m, 2H, H2+H5), 1.60 (br. quin, J = 6.8 Hz, 4H, CH_2, dodecyl), 1.22–1.38 (m, 36H, CH_2, dodecyl), 0.88 (t, J = 6.7 Hz, 6H, CH_3). ^{13}C NMR (75 MHz, $CDCl_3$): 172.47, 64.84, 50.88, 37.62, 31.90, 29.63, 29.62, 29.57, 29.52, 29.32, 29.24, 28.53, 25.95, 24.94, 22.65, 14.05. HRMS: $C_{32}H_{58}O_5$ requires *m/z* $[M+Na]^+$ 545.4177, found 545.4164.

*Didodecyl trans-4-hydroxy-cis-5-morpholylcyclohexane-cis-1,2-dicarboxylate (**2**)*

anti-Epoxide **5a** (1.0 g, 1.9 mmol) and morpholine (0.9 mL, 10 mmol) were stirred 15 h at 40 °C in 5 mL of iPrOH:H_2O (1.5:1). The reaction mixture was concentrated on rotary evaporator, and the product **2** was isolated as a clear oil by silica gel column chromatography (hexane:EtOAc, 2:1): yield 0.77 g (66%). ^{1}H NMR (600 MHz, CD_3OD): 4.06 (m, 4H, OCH_2, dodecyl), 3.68 (m, 4H, OCH_2, morpholyl), 3.54 (dt, J = 4.3, 10.4 Hz, 1H, H4), 3.25 (br. q, J = 4.2 Hz, 1H, H2), 2.70 (m, 2H, NCH_2, morpholyl), 2.64 (dt, J = 12.3, 4.2 Hz; 1H, H1), 2.52 (m, 2H, NCH_2, morpholyl), 2.41 (ddd, J = 13.2, 4.2, 3.2 Hz; 1H, H3e), 2.33 (ddd, J = 12.1, 9.8, 3.8 Hz; 1H, H5), 2.14 (ddt, J = 13.4, 1.2, 3.7 Hz; 1H, H6e), 1.84 (q, J = 12.6 Hz; 1H, H6a), 1.62 (q, J = 12 Hz; 1H, H3a), 1.61 (m, 4H, CH_2, dodecyl), 1.24–1.39 (m, 36H, CH_2, dodecyl), 0.90 (t, J = 7.0 Hz; 6H, CH_3). ^{13}C NMR (150 MHz, CD_3OD): 174.65, 174.61, 69.55, 68.40, 66.45, 65.91, 50.26, 43.58, 42.31, 35.43, 33.13, 30.87, 30.83, 30.80, 30.77, 30.55, 30.44, 29.81, 29.68, 27.17, 23.79, 22.95, 14.51. HRMS: $C_{36}H_{67}NO_6$ requires $[M+H]^+$ 610.5041, found 610.5032. MS/MS *m/z* (rel. intensity): 201.5 (5), 238.1 (13), 256.12 (100), 378.3 (11),

424.1 (85), 442.3 (27), 592.5 (90), 610.5 (8) $[M+H]^+$. Calcd. for $C_{36}H_{67}NO_6$: C, 70.89; H, 11.07; N, 2.30. Found: C, 71.10; H, 11.84; N, 2.27.

*Didodecyl cis-4-hydroxy-trans-5-morpholylcyclohexane-cis-1,2-dicarboxylate (**3**)*

syn-Epoxide **5s** (0.85 g, 1.6 mmol) was reacted with morpholine the same way as described above to yield 0.59 g **3** (60%). ^{1}H NMR (600 MHz, CD_3OD): 4.06 (m, 4H, OCH_2, dodecyl), 3.68 (m, 4H, OCH_2, morpholyl), 3.60 (dt, J = 4.7, 10.3 Hz, 1H, H4), 3.31 (m, 1H, H1; overlapped with OH), 2.71 (m, 2H, NCH_2, morpholyl), 2.64 (dt, J = 12.8, 4.2 Hz; 1H, H2), 2.49 (m, 2H, NCH_2, morpholyl), 2.32 (ddt, J = 13.1, 1.3, 4.1 Hz; 1H, H3e), 2.29 (dt, J = 13.3, 3.2 Hz; 1H, H6e), 2.24 (ddd, J = 12.2, 9.8, 3.3 Hz; 1H, H5), 1.80 (dt, J = 11.2, 12.8 Hz; 1H, H3a), 1.63 (m, 1H, H6a), 1.61 (m, 4H, CH_2, dodecyl), 1.24–1.40 (m, 36H, CH_2, dodecyl), 0.90 (t, J = 7.0 Hz; 6H, CH_3). ^{13}C NMR (150 MHz, CD_3OD): 174.58, 174.48, 68.94, 68.39, 67.03, 65.97, 65.90, 50.12, 42.80, 42.48, 42.40, 33.12, 30.85, 30.83, 30.81, 30.76, 30.54, 30.45, 30.42, 29.85, 29.65, 27.23, 27.16, 25.59, 23.78, 14.48. HRMS: $C_{36}H_{67}NO_6$ requires $[M+H]^+$ 610.5041, found 610.5035. MS/MS *m/z* (rel. intensity): 140.5 (13), 169.0 (9), 187.5 (13), 238.4 (9), 255.6 (16), 376.9 (5), 424.3 (7), 591.9 (16), 610.1 (100) $[M+H]^+$.

Reference

1. Brazdova, B.; Zhang, N.; Samoshin, V.V.; Guo, X. *trans*-2-Aminocyclohexanol as a pH-sensitive conformational switch in lipid amphiphiles. *Chem. Commun.* **2008**, *39*, 4774-4776.
